# Hepatitis E Virus Infection Epidemiology in Recipients of Allogeneic Hematopoietic Cell Transplant

**DOI:** 10.1093/ofid/ofad595

**Published:** 2023-11-24

**Authors:** Johan Courjon, Vera Portillo, Sabine Yerly, Pauline Vetter, Manuel Schibler, Maria Mappoura, Sarah Morin, Federica Giannotti, Anne-Claire Mamez, Christian van Delden, Laurent Kaiser, Yves Chalandon, Stavroula Masouridi-Levrat, Dionysios Neofytos

**Affiliations:** Division of Infectious Diseases, University Hospital of Geneva, Geneva, Switzerland; Université Côte d’Azur, Inserm, C3M, Nice, France; Division of Infectious Diseases, University Hospital of Geneva, Geneva, Switzerland; Laboratory of Virology, Division of Laboratory Medicine, University Hospital of Geneva, Geneva, Switzerland; Division of Infectious Diseases, University Hospital of Geneva, Geneva, Switzerland; Laboratory of Virology, Division of Laboratory Medicine, University Hospital of Geneva, Geneva, Switzerland; Geneva Center for Emerging Viral Diseases, Geneva University Hospitals, Geneva, Switzerland; Division of Infectious Diseases, University Hospital of Geneva, Geneva, Switzerland; Laboratory of Virology, Division of Laboratory Medicine, University Hospital of Geneva, Geneva, Switzerland; Division of Hematology, Bone Marrow Transplant Unit, University Hospital of Geneva and Faculty of Medicine, University of Geneva, Geneva, Switzerland; Division of Hematology, Bone Marrow Transplant Unit, University Hospital of Geneva and Faculty of Medicine, University of Geneva, Geneva, Switzerland; Geneva Center for Emerging Viral Diseases, Geneva University Hospitals, Geneva, Switzerland; Division of Hematology, Bone Marrow Transplant Unit, University Hospital of Geneva and Faculty of Medicine, University of Geneva, Geneva, Switzerland; Division of Infectious Diseases, University Hospital of Geneva, Geneva, Switzerland; Division of Infectious Diseases, University Hospital of Geneva, Geneva, Switzerland; Division of Hematology, Bone Marrow Transplant Unit, University Hospital of Geneva and Faculty of Medicine, University of Geneva, Geneva, Switzerland; Division of Hematology, Bone Marrow Transplant Unit, University Hospital of Geneva and Faculty of Medicine, University of Geneva, Geneva, Switzerland; Division of Infectious Diseases, University Hospital of Geneva, Geneva, Switzerland

**Keywords:** acute hepatitis E, allogeneic hematopoietic cell transplantation, hepatitis E virus, immunosuppression, seroprevalance

## Abstract

Among 292 recipients of allogeneic hematopoietic cell transplant (2018–2022), 64 (21.9%) tested positive for anti–hepatitis E virus (HEV) immunoglobulin G. Among 208 recipients tested by plasma/serum HEV polymerase chain reaction (2012–2022), 3 (1.4%) primary HEV infections were diagnosed; in 1 patient, plasma HEV polymerase chain reaction relapsed positive for 100 days. HEV infection remains rare albeit associated with persistent viral replication.

Hepatitis E virus (HEV) infection is a frequent and often unrecognized infection with varying epidemiologic patterns across different regions. Once infected with HEV, patients who are immunocompromised, including recipients of solid organ transplant and hematopoietic cell transplant (HCT), are prone to develop protracted infections with increased rates of morbidity and mortality [1, 2]. Limited data exist on the seroprevalence and incidence of HEV in allogeneic HCT recipients, with a reported prevalence ranging from <1% to 4% [3]. In this study we describe the epidemiology and outcomes of HEV infection in a contemporary cohort of allogeneic HCT recipients.

## METHODS

This is an observational retrospective single-center cohort study conducted at a Swiss university hospital, including consecutive adult (≥18 years old) allogeneic HCT recipients. The study was approved by the local ethics committee (2020-02120). The objectives of this study were to describe the seroprevalence of HEV and the incidence of HEV infection posttransplant in a cohort of allogeneic HCT recipients.

### Seroprevalence in Patients With Infectious Disease Assessment Before Transplantation

Between 1 January 2018 and 31 December 2022, HEV serology testing was consistently performed in all adult allogeneic HCT recipients in the context of a pretransplant infectious disease consultation (V Portillo et al, manuscript under revision). Patients were tested for anti-HEV immunoglobin G (IgG) with an enzyme-linked immunosorbent assay (Wantai Biopharmaceutical), according to the manufacturer's instructions. All sera with a low level of anti-HEV IgG (index, 1.0–10) were confirmed by immunoblot assay (RecomLine; Mikrogen).

### Incidence of HEV Infection

HEV RNA detection in plasma and stools was performed in allogeneic HCT recipients upon clinical suspicion, mainly in cases of acute hepatitis, via an in-house reverse transcriptase polymerase chain reaction (RT-PCR) assay, as previously described [4]. Since October 2018, plasma samples were tested for HEV RNA with the Cobas HEV assay on the Cobas 6800 platform (Roche Diagnostics). Patients with a positive HEV RT-PCR test result were identified by the virology laboratory during an arbitrarily selected 10-year period between 2012 and 2022. Data on the diagnosis and management of HEV infections were retrospectively collected from the electronic health record of each patient.

### Definitions

HEV infection was defined as an increase of liver enzymes with a positive plasma HEV RT-PCR test result and no other possible etiologies. As previously reported, patients with HEV RNA detection for at least 3 months were considered as having a protracted HEV infection [3].

### Data Collection

Data on demographics and HCT-associated variables were collected in the institutional bone marrow transplant database. The following additional data were collected through electronic chart review: HEV serologic status and plasma RT-PCR results, as well as the time, clinical presentation, and treatment of HEV infection.

### Statistical Analyses

Continuous variables were expressed as mean and SD or median and IQR and categorial variables as number and percentage. Comparisons were done through a Fisher exact test for categorical variables and a Student *t* test or Mann-Whitney *U* test for continuous variables, if required. All data were analyzed with Prism software (GraphPad, Version 9).

## RESULTS

### HEV Seroprevalence

Among 292 patients who underwent transplantation between 2018 and 2022 with a median follow-up of 17 months (IQR, 24) posttransplant, 66 (22.6%) tested positive for anti-HEV IgG at the time of their pretransplant evaluation; of these, 64 (97%) were confirmed positive by immunoblot. The baseline characteristics of those patients are presented in Table 1. When compared with patients who were HEV seronegative, patients who were HEV seropositive were older (60.8 vs 53.1 years, *P* < .001) and more likely to demonstrate a cytomegalovirus donor^−^/recipient^+^ serology constellation (28.1% vs 16.2%, *P* = .04). There were no significant differences in underlying disease, stem cell source, donor type, conditioning, graft-vs-host disease (GvHD) occurrence, or travel history between the groups.

**Table 1.** Characteristics of 292 Allogeneic HCT Recipients According to HEV Serology Before Transplantation

	HCT Recipients, No. (%)			
	All (n = 292)	HEV Seropositive (n = 64)	HEV Seronegative (n = 228)	*P* Value
Demographics				
** **Age, y, mean (SD)	55 (13.8)	60.8 (10.2)	53.1 (14.3)	<.001
** **Sex, male	186 (63.4)	40 (62.5)	146 (64)	.82
Origin^a^				
** **Europe	185 (87.3)	41 (87.2)	144 (86.7)	>.99
** **Africa	12 (5.6)	4 (6.1)	8 (4.8)	.30
** **Asia	11 (5.1)	3 (4.5)	8 (4.8)	.71
** **South/Central America	5 (2.3)	1 (1.5)	4 (2.4)	>.99
Travel outside Europe^b^	184 (68.7)	42 (77.2)	142 (67.9)	.75
Underlying disease				
** **AML/MDS	182 (62.3)	39 (60.9)	143 (62.7)	.88
** **Lymphoma	35 (11.9)	4 (6.2)	31 (13.6)	.13
** **ALL	20 (6.8)	7 (10.9)	13 (5.8)	.16
** **CML/CLL	16 (5.5)	4 (6.2)	12 (5.7)	.76
** **Other^c^	39 (13.3)	10 (15.6)	29 (12.7)	.54
Cytomegalovirus status				
** **D−/R−	86 (29.5)	17 (26.5)	69 (30.3)	.64
** **D+/R+	125 (42.8)	25 (39.1)	100 (43.8)	.57
** **D+/R−	26 (8.9)	4 (6.2)	22 (9.6)	.47
** **D−/R+	55 (18.8)	18 (28.1)	37 (16.2)	.04
HCT-associated variables				
** **Stem cell source				
** **PBSCs	270 (92.5)	60 (93.9)	210 (92.1)	.79
** **Bone marrow	22 (7.5)	4 (6.1)	18 (7.9)	.79
** **Donor type				
** **MRD	61 (20.9)	11 (16.7)	50 (22.1)	.49
** **MUD	130 (44.5)	31 (47)	99 (43.8)	.48
** **MMUD	21 (7.2)	4 (6.1)	17 (7.5)	>.99
** **Haploidentical	80 (27.4)	20 (30.3)	60 (26.5)	.43
** **Conditioning, RIC	223 (76.4)	54 (84.3)	169 (74.1)	.1
** **Time to engraftment, d, mean (SD)	18.9 (4.4)	19.8 (4.1)	18.5 (4.4)	.12
** **GvHD ≥2	124 (42.5)	25 (39.1)	99 (43.4)	.57
Follow-up since HCT, mo, mean (SD)	20.3 (15.4)	20.7 (17.2)	20.2 (14.8)	.80

Abbreviations: ALL, acute lymphoblastic leukemia; AML, acute myeloid leukemia; CLL, chronic lymphocytic leukemia; CML, chronic myeloid leukemia; D−, donor negative; D+, donor positive; GvHD, graft-vs-host disease; HCT, hematopoietic cell transplant; HEV, hepatitis E virus; MDS, myelodysplastic syndrome; MMUD, mismatched unrelated donor; MRD, matched related donor; MUD, matched unrelated donor; PBSC, peripheral blood stem cell; R−, recipient negative; R+, recipient positive; RIC, reduced-intensity conditioning.

^a^Information available for 73% (213/292) of the patients.

^b^Information available for 92% (268/292) of the patients.

^c^Other: multiple myeloma, hemoglobinopathy, myeloproliferative syndrome.

### Incidence of Active HEV Infection

**Figure 1.**
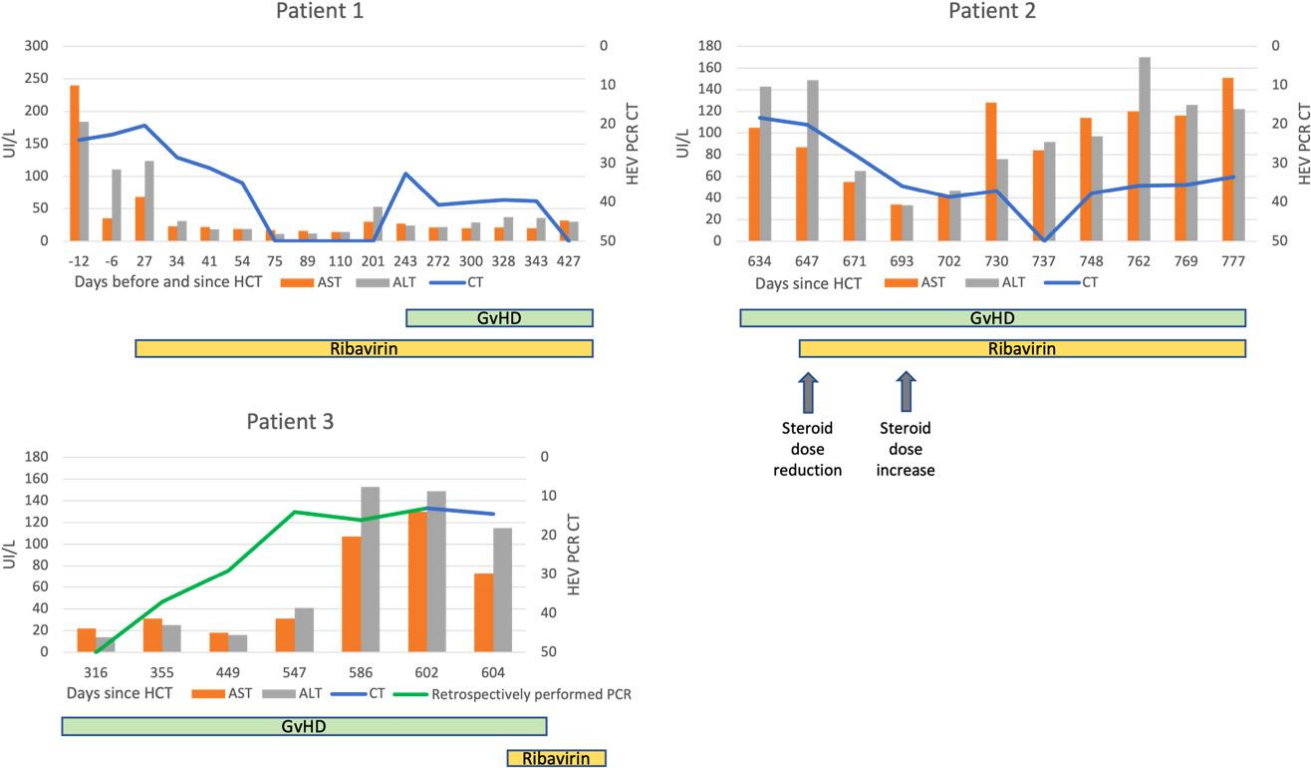
Characteristics of 3 recipients of an allogeneic HCT with acute HEV infection posttransplant, with evolution of liver function tests and Ct levels of HEV RT-PCR depicted with GvHD and ribavirin treatment periods. For each time point represented on graphs by days before and after transplant, a plasma HEV RT-PCR was performed. ALT, alanine aminotransferase; AST, aspartate aminotransferase; Ct, cycle threshold; GvHD, graft-vs-host disease; HCT, hematopoietic cell transplant; HEV, hepatitis E virus; RT-PCR, reverse transcriptase polymerase chain reaction. Patient 1 was a 34-year-old allogeneic HCT recipient with extranodal NK/T-cell lymphoma previously treated with an autologous HCT. He was diagnosed with HEV infection based on elevated liver enzymes (AST, 240 UI/L; ALT, 184 UI/L; γ-glutamyltransferase, 13 UI/L; total bilirubin, 31 μmol/L) without any clinical symptoms 12 days before the allogeneic HCT procedure. Consumption of raw sea food within several weeks before diagnosis was identified as the major risk factor. HEV RNA was detected in plasma and stools upon diagnosis, with Ct values of 24 and 34, respectively. Treatment with ribavirin was initiated orally at a dose of 600 mg/d 34 days after diagnosis. The first negative plasma RT-PCR result was recorded after 55 days of treatment initiation, 89 days after diagnosis. Treatment was continued for 918 days, until death occurred because of septic shock with lymphoma relapse. Under treatment, HEV RNA in plasma was not detected between 75 and 201 days posttransplant. A transient viral rebound was observed between 243 and 343 days posttransplant in the context of newly diagnosed GvHD (oral, genital, and skin) treated with high-dose corticosteroids. HEV RNA was again undetectable after 427 days posttransplant and remained negative until death. Patient 2 was 60 years old with multiple myeloma previously treated with an autologous HCT. The patient was diagnosed with liver GvHD 8 months posttransplant, 1 year prior to the diagnosis of HEV infection. HEV RNA was not detected in plasma at the time of liver GvHD diagnosis. The patient developed elevated liver enzymes (AST, 105 UI/L; ALT, 143 UI/L; γ-glutamyltransferase, 4351 UI/L; total bilirubin, 6 μmol/L) without symptoms, which prompted a test for HEV RNA in plasma 20 months after allogeneic HCT. The RT-PCR test result was positive with a Ct value of 18.3. HEV RNA was also detected in stools upon diagnosis (Ct, 22.6). No specific exposure could be identified. Treatment with oral ribavirin at a dose of 400 mg/d was initiated 12 days after the diagnosis. Sixty-six days later, no HEV RNA could be detected in plasma. HEV RNA was again positive with high Ct values (33.5-37.7) until death, 143 days after treatment initiation. The patient died of multiorgan failure with adenovirus systemic infection, norovirus enteritis, and polymicrobial bacteremia in the context of skin, intestinal, and hepatic GvHD. Patient 3 was a 23-year-old allogenic HCT recipient with anaplastic large cell lymphoma previously treated with an autologous HCT. The patient was diagnosed with steroid-refractory GvHD of the gastrointestinal tract 1 month posttransplant and disseminated aspergillosis 574 days posttransplant. After antifungal initiation, he developed elevated liver enzymes (AST, 130 UI/L; ALT, 149 UI/L; γ-glutamyltransferase, 610 UI/L; total bilirubin, 11 μmol/L). Drug-induced liver injury was considered, but HEV RNA was also detected in plasma with a Ct value of 13, 602 days posttransplant. Treatment with oral ribavirin at a dose of 600 mg/d was initiated 7 days after the diagnosis and stopped 5 days later because of pancreatitis. He died 697 days posttransplant because of an invasive fungal infection. Notably, HEV RT-PCR tests were retrospectively performed, and HEV RNA could be detected up to 247 days before HEV infection diagnosis without elevated liver enzymes at this time.

Between 2012 and 2022, 699 patients underwent at least 1 allogeneic HCT, with a median follow-up of 23 months (IQR, 48) posttransplant. Among them, 352 HEV RT-PCR tests on plasma/serum were requested in 208 (29.8%) patients. Overall, 20 (5.7%) tests were positive in 3 (1.4%) tested patients, who developed chronic HEV infection (1 in 2016 and 2 in 2018). All 3 patients had a negative HEV serology test result before their allogeneic HCT and presented with abnormal liver function test results. Genotyping was unavailable in all 3 cases. Anti-HEV serology was repeated in only 1 patient and remained negative until death. Details on the clinical presentation and course of those patients—including liver function tests, plasma HEV RNA cycle threshold values, information on posttransplant complications (eg, GvHD), and antiviral treatment—are presented in Figure 1.

## DISCUSSION

In this contemporary European single-center cohort of allogeneic HCT recipients, we report an HEV seroprevalence of 21.9% prior to transplant. Primary HEV infection was diagnosed in only 3 (1.4%) of 208 patients tested (all seronegative before transplantation), reflecting a relatively low risk to acquire this infection after transplantation. Our findings highlight the frequency of HEV infection in the general population and the importance of clinical vigilance in high-risk cases, such as allogeneic HCT recipients. In 2 of the 3 HEV infections, poor virologic control was documented during immunosuppression associated with GvHD management, even with ribavirin treatment.

The 21.9% HEV seroprevalence found in our cohort is consistent with the 22.3% reported in the general population of Switzerland [5]. Higher age was associated with seropositivity, suggesting a potential cumulative effect of age on the risk of HEV infection. In contrast, HEV IgG seroprevalence in Italian HCT recipients (92.4% of allogeneic HCT) from 2010 to 2019 was much lower: 6% (34/563) before transplantation [6]. With those previously published data, our results suggest that HEV seroprevalence highly varies across countries. Such discrepancy may be due to different exposure to risk factors, such as food consumption habits, but also to the performance of the serologic test used. Blood products administered in high-risk hematology cases may contain exogenous IgG, potentially detected by serology tests. Conversely, lower sensitivity in serology tests in solid organ transplant recipients and false-negative serology results in HCT recipients have been reported [7, 8].

Different works have emphasized the lack of association between previous HEV exposure and HEV infection after transplantation [9, 10]. Otherwise, in a landmark case report of HEV infection in the context of allogeneic HCT, le Coutre et al described a patient with chemotherapy-induced cytopenia and a diagnosis of HEV infection 63 days before transplantation [11]. Although HEV RT-PCR became negative 28 days before transplantation, viral RNA was detected again 56 days posttransplant. After review of the existing body of evidence on the outcomes of HEV infections in 52 HCT recipients in 5 studies, 4 (7.7%) rebound HEV infections have been described [3, 10, 12–14]. Three of those were detected 1 to 4 months after the first negative HEV RT-PCR test result in plasma in the absence of ribavirin treatment, and 1 was documented 34 days after completion of a 6-month ribavirin treatment. There have been no definitive cases of HEV reactivation (despite being labeled as such), although cases of HEV viremia rebound after viral clearance and reinfection have been reported [15]. Indeed, RNA viruses such as HEV are not known to establish latency and therefore nor to reactivate [16]. However, those viruses may lead to persistent infection, particularly in individuals who are immunocompromised, whose immune system is unable to fully control the initial acute infection. Notably, in a study by Mikulska et al on allogeneic HCT recipients, HEV RNA clearance occurred in 85% of cases [3]. This clearance was significantly reduced in patients receiving immunosuppression at the time of HEV diagnosis, and immunosuppression reduction led to reduced time to HEV clearance [3]. Those data and ours suggest that allogeneic HCT recipients with HEV infection may not always be able to fully clear the virus at the time of primary infection, particularly when it occurs during periods of important immunosuppression. They remain at higher risk to develop persistent infection, the control of which predominately depends on their immune reconstitution rather than the administration of antiviral treatment with ribavirin.

This study reports on the incidence and prevalence of HEV infections in one of the largest allogeneic HCT cohorts published today. Repeated HEV RNA testing in plasma allowed an accurate description of HEV infection evolution. Phylogenetic analyses were not performed. Genotyping was not performed in any of the 3 cases of HEV infection reported, and none of those patients had traveled outside of Switzerland posttransplant. However, almost all HEV strains isolated from patients with acute hepatitis acquired in Switzerland have been genotype 3 [17]. Finally, the discrepancy between high pretransplant prevalence and low posttransplant incidence may be in part due to the higher degree of awareness and more prudent eating habits of HCT recipients posttransplant and/or the biases associated with less frequent testing and lower clinical suspicion. In fact, testing plasma HEV RNA was not performed in all recipients posttransplant but only on a clinical basis when a diagnostic procedure was required in case of elevated liver enzymes. Although this could have prevented us from detecting asymptomatic primary HEV infection in the posttransplant setting—a common manifestation in patients who are immunocompetent and in recipients of solid organ transplant—most HEV infection in allogeneic HCT recipients has been associated with abnormal liver function test results [10, 18, 19]. Nevertheless, to avoid potential underreporting biases, we have described the rate of new HEV infection over the number of patients tested and not the overall number of patients who underwent transplantation during the study period.

## CONCLUSIONS

Despite a relatively high seroprevalence, HEV infection remains rare among HCT recipients. Primary infection during periods of high immunosuppression posttransplant may be associated with persistent viral replication and chronic infection, despite administered antiviral treatment.

## Notes

***Acknowledgments.*** We thank the nursing personnel and all our patients in the Bone Marrow Transplant Unit and the virology laboratory of our institution.

***Author contributions.*** J. C., V. P., and D. N. designed the study. J. C., V. P., S. Y., P. V., M. S., M. M., S. M., F. G., A.-C. M., C. v. D., L. K., Y. C., S. M.-L., and D. N. collected the data. J. C., V. P., S. Y., P. V., L. K., Y. C., and D. N. interpreted the data. J. C., V. P., S. Y., P. V., L. K., Y. C., and D. N. wrote the manuscript.

***Patient consent statement.*** All patients included in this study had signed an informed consent form upon admission for their transplantation to allow the use of their clinical data for clinical research. The study was approved by the Ethics Committee of the University of Geneva (2020-02120).
